# An exploratory study of the effects of spatial working-memory load on prefrontal activation in low- and high-performing elderly

**DOI:** 10.3389/fnagi.2014.00303

**Published:** 2014-11-05

**Authors:** Anouk Vermeij, Arenda H. E. A. van Beek, Babette L. R. Reijs, Jurgen A. H. R. Claassen, Roy P. C. Kessels

**Affiliations:** ^1^Donders Institute for Brain, Cognition and Behaviour, Radboud University NijmegenNijmegen, Netherlands; ^2^Department of Geriatric Medicine, Radboud University Medical CenterNijmegen, Netherlands; ^3^Department of Psychiatry and Neuropsychology, Maastricht UniversityMaastricht, Netherlands; ^4^Department of Medical Psychology, Radboud University Medical CenterNijmegen, Netherlands

**Keywords:** BOLD, cognitive aging, CRUNCH, functional near-infrared spectroscopy, HAROLD, n-back, prefrontal cortex, working memory

## Abstract

Older adults show more bilateral prefrontal activation during cognitive performance than younger adults, who typically show unilateral activation. This over-recruitment has been interpreted as compensation for declining structure and function of the brain. Here we examined how the relationship between behavioral performance and prefrontal activation is modulated by different levels of working-memory load. Eighteen healthy older adults (70.8 ± 5.0 years; MMSE 29.3 ± 0.9) performed a spatial working-memory task (n-back). Oxygenated ([O_2_Hb]) and deoxygenated ([HHb]) hemoglobin concentration changes were registered by two functional Near-Infrared Spectroscopy (fNIRS) channels located over the left and right prefrontal cortex. Increased working-memory load resulted in worse performance compared to the control condition. [O_2_Hb] increased with rising working-memory load in both fNIRS channels. Based on the performance in the high working-memory load condition, the group was divided into low and high performers. A significant interaction effect of performance level and hemisphere on [O_2_Hb] increase was found, indicating that high performers were better able to keep the right prefrontal cortex engaged under high cognitive demand. Furthermore, in the low performers group, individuals with a larger decline in task performance from the control to the high working-memory load condition had a larger bilateral increase of [O_2_Hb]. The high performers did not show a correlation between performance decline and working-memory load related prefrontal activation changes. Thus, additional bilateral prefrontal activation in low performers did not necessarily result in better cognitive performance. Our study showed that bilateral prefrontal activation may not always be successfully compensatory. Individual behavioral performance should be taken into account to be able to distinguish successful and unsuccessful compensation or declined neural efficiency.

## Introduction

Studies on the cognitive neuroscience of aging have reliably revealed age-related differences in brain activation during cognitive task performance (for reviews, see Spreng et al., 2010; Eyler et al., 2011; Grady, 2012; Turner and Spreng, 2012). Two patterns of age-related differences in brain activation have been consistently reported. The first is an age-related reduction in occipitotemporal activation together with an age-related increase in activation of the prefrontal cortex. This has been called the “posterior-anterior shift in aging” (PASA; Grady et al., 1994; Davis et al., 2008). The second is a more bilateral pattern of prefrontal activation in older adults on tasks for which young adults typically show unilateral activation. This pattern has been referred to as Hemispheric Asymmetry Reduction in OLDer adults (HAROLD; Cabeza, 2002). Age-related over-recruitment of the prefrontal cortex has been observed across several cognitive domains such as perception, attention, memory encoding and retrieval, and executive functioning, but most extensively for working memory and inhibitory control tasks (Spreng et al., 2010).

Over-recruitment of the prefrontal cortex in older adults has been interpreted as a compensatory mechanism that can aid cognitive performance (Cabeza, 2002). The traditional cognitive aging theories, such as the sensory deficit theory (Baltes and Lindenberger, 1997), resources deficit theory (Craik, 1986), speed deficit theory (Salthouse, 1996), and inhibition deficit theory (Hasher and Zacks, 1988) were developed to explain age-related differences in behavioral performance, but did not always incorporate assumptions regarding age-related differences in brain activation. Dennis and Cabeza (2008) expanded these traditional theories with additional assumptions regarding brain correlates of relevant cognitive processes and regarding compensatory mechanisms. They concluded that these theories are consistent with the notion of compensation and with evidence from functional neuroimaging studies. Alternatively, age-related prefrontal over-recruitment may reflect less efficient use of neural resources or a less selective recruitment of brain areas, also known as dedifferentiation, which might not necessarily lead to better task performance (Logan et al., 2002). Although there is support for both alternatives, most neuroimaging results are consistent with the compensation account rather than the dedifferentiation account (Spreng et al., 2010; Eyler et al., 2011).

Meta-analysis of performance-related prefrontal activation across cognitive domains revealed that when performance was equivalent in young and older adults, young adults showed stronger activity in the left ventrolateral prefrontal cortex, whereas older adults showed stronger activity in the left dorsolateral prefrontal cortex. When performance was not equivalent, worse performing older adults showed stronger recruitment of the right dorsolateral prefrontal cortex and right rostrolateral prefrontal cortex (Spreng et al., 2010). The meta-analytic review by Turner and Spreng (2012) provided evidence that patterns of age-related functional brain change are dissociable for two of the most frequently studied executive processes: inhibition and working memory. During inhibitory control tasks, older adults engaged brain regions commonly recruited in younger adults, but to a larger extent. In contrast, during working-memory performance, older adults showed stronger recruitment of both left and right dorsolateral prefrontal cortex than younger adults. These results were consistent with previous studies on working memory reporting larger and less lateralized recruitment of the dorsolateral prefrontal cortex (Reuter-Lorenz et al., 2000; Cabeza et al., 2004). Due to lack of sufficient statistical power, the relationship between performance differences and prefrontal activation patterns could unfortunately not be examined in the meta-analytic review by Turner and Spreng (2012).

An unresolved issue is how over-recruitment of the prefrontal cortex is associated with the variation of cognitive performance levels among older adults. The review by Eyler et al. (2011) focused on the association between functional response and cognitive performance in healthy young and older adults. Of the 74 reviewed studies that examined the relation between prefrontal activation and cognitive performance, 35% found a positive correlation, 18% found a negative correlation, 16% found mixed results and 31% did not find a significant correlation. Of the 29 studies that were consistent with HAROLD and/or PASA patterns, 34% found a positive correlation between prefrontal activation and cognitive performance, whereas 27% found a negative correlation. Although these results suggest that increased prefrontal activation might be beneficial rather than detrimental at older age, clearly more work is needed to unravel the brain-behavior correlations at older age.

The aim of the present study is to gain more insight into the role of over-recruitment of the prefrontal in older adults during working-memory performance. Specifically, we examined the relationship between prefrontal activation and behavioral performance by comparing high and low performers. The participants performed a spatial working-memory task with varying levels of cognitive load while their prefrontal activation was measured by functional Near-Infrared Spectroscopy (fNIRS). fNIRS enables monitoring of concentration changes of oxygenated hemoglobin ([O_2_Hb]) and deoxygenated hemoglobin ([HHb]) in the cortex with high temporal resolution. In comparison to fMRI, fNIRS has the advantage that it is less expensive, less invasive, less sensitive to movement artefacts, and that it is portable. The spatial resolution of fNIRS is however limited (Cui et al., 2011; Ferrari and Quaresima, 2012). The majority of neuroimaging studies investigating the brain-behavior relationship in older adults assessed cognitive performance by accuracy, followed by reaction time (Eyler et al., 2011). In the current study, cognitive performance was assessed by a composite score of these measures to take speed/accuracy trade-offs into account and to diminish strategy effects.

Prefrontal activation is modulated by working-memory load. Previous fMRI studies showed that in young as well as older adults, prefrontal activation increases with working-memory load up to where the working-memory capacity limit is reached, and then levels off or decreases (Mattay et al., 2006; Schneider-Garces et al., 2009). In order to explain contrasting evidence of both age-related under-recruitment as well as age-related over-recruitment of the prefrontal cortex during working-memory performance, Reuter-Lorenz and Cappell (2008) formulated the Compensation-Related Utilization of Neural Circuits Hypothesis (CRUNCH). CRUNCH proposes that, irrespective of age, neural engagement varies with the level of task demand; activity in cortical regions is upregulated up to a certain level as cognitive load increases. The relationship between cognitive load and brain activation has been described as an S-shaped function. The idea behind CRUNCH is that at low levels of cognitive load, older adults need to recruit more neural resources than young adults in order to maintain task performance, due to less efficient neural processing at older age. At high levels of cognitive load, this compensatory mechanism is no longer effective, leading to reduced or equivalent activation in older adults in comparison to young adults. Hence, in older adults the S-shaped function would be shifted to the left relative to young adults. It has been proposed that a similar effect would be observed when low-performing older adults are compared to high-performing older adults (Grady, 2012), resulting in a leftward shift of the S-shaped curve in low performers relative to high performers. Therefore, in the current study, we expected that high-performing older adults would show increasing prefrontal activation up to a high level of working-memory load. Furthermore, we hypothesized that low performers would reach their working-memory capacity limit sooner than high performers, reflected by reduced prefrontal activation in low performers compared to high performers at a high level of working-memory load.

## Materials and methods

### Participants

Eighteen healthy older adults participated in this study. Sample characteristics are shown in Table 1. All participants had completed secondary school or higher. Estimated IQ was based on assessment of the Dutch equivalent of the National Adults Reading Test (Schmand et al., 1992). None of the older adults experienced subjective memory problems, all were living independently at home, and all had unimpaired overall cognitive function as assessed with the Mini Mental State Examination (MMSE; Folstein et al., 1975). All participants were right-handed and had normal or corrected-to-normal vision. None of the participants had a history of neurological or psychiatric disease, or received psychopharmacological drugs or hormone therapy (self report). Six participants used antihypertensive medication. All participants refrained from alcohol, caffeine, and nicotine from at least 3 h before the experimental session. The research proposal for the present study was submitted to the regional medical-ethics committee (CMO Arnhem-Nijmegen, no. 2009/198), but was deemed exempt from formal medical ethical evaluation, because the study does not fall within the remit of the Medical Research Involving Human Subjects Act (WMO). All participants gave written informed consent. The study was performed according to the Helsinki Declaration.

**Table 1:** **Sample characteristics**.

	Total group	Low performers	High performers
Participants	18 (11 female, 7 male)	9 (6 female, 3 male)	9 (5 female, 4 male)
Age	70.8 ± 5.0 (range 64–81)	72.7 ± 5.6 (range 64–81)	69.0 ± 3.8 (range 65–77)
Years of education	13.1 ± 3.2 (range 9–18)	13.5 ± 3.4 (range 9–18)	12.7 ± 3.1 (range 9–18)
Estimated IQ	108.1 ± 11.0 (range 87–124)	109.7 ± 11.3 (range 90–124)	106.4 ± 11.0 (range 87–118)
MMSE	29.3 ± 0.9 (range 27–30)	29.7 ± 0.7 (range 28–30)	28.9 ± 0.9 (range 27–30)

### Experimental procedure

**Figure 1:**
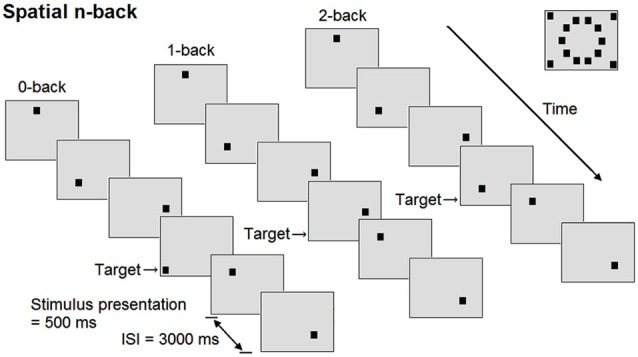
**Schematic overview of the spatial n-back task**. At the right upper corner of the figure, all possible positions of the square are shown. ISI = interstimulus interval.

Participants performed three versions of a spatial n-back task (Figure 1): 0-back task (control condition), 1-back (low working-memory load), and 2-back task (high working-memory load condition). Prior to all conditions, participants practiced the task for 1 min and received feedback about their performance. The conditions were presented in ascending order and were preceded by a baseline period of 1 min, during which a black fixation cross was displayed at the center of the 15 inch screen. All conditions consisted of 60 trials, 17 of which were target trials. In each trial, a square was presented in black on a light gray background with a presentation time of 500 ms at 1 of 14 pre-specified locations on the display. During the interstimulus interval of 3000 ms, a fixation cross was displayed. During each trial, participants indicated whether the stimulus was a target by pressing the button under the right index finger, or a non-target by pressing the button under the right middle finger (PST Serial Response Box, Psychology Software Tools Inc., PA, USA). Participants were allowed to respond until the next stimulus appeared. In the 0-back condition, a square at one of the four outer corners of the screen was defined as target. In the 1-back condition, the target was any square that appeared at the same location as the square presented one trial before, while squares no longer appeared in the corners. In the 2-back condition, the target was any square that appeared at the same location as the square presented two trials before. In order to prevent verbalization of the locations by the participant, no grid or clock configuration of the squares was chosen. The experimental procedure lasted around 20 min per participant. To minimize effects of fatigue, participants were able to rest a couple of minutes between conditions.

### Data acquisition

We used a continuous-wave NIRS device (Oxymon Mk III, Artinis Medical Systems, Netherlands), using light of three wavelengths (765, 857, 859 nm), to monitor concentration changes in cortical [O_2_Hb] and [HHb] with high temporal resolution. The principle behind fNIRS is that near-infrared light penetrates the skull and brain and is absorbed by the chromophores [O_2_Hb] and [HHb], which have different absorption spectra (Ferrari and Quaresima, 2012). Assuming constant scattering (Sakatani et al., 2006), and by using the modified Lambert-Beer Law, it is possible to calculate the concentration changes of these chromophores in the penetrated brain tissue based on changes in the detected light intensity. Increases in [O_2_Hb] and decreases in [HHb] are indicators of cortical activation.

In the present study, two pairs of optodes were bilaterally attached to the forehead and were tightly fixed in a customized headband (Spencer technologies, Seattle, WA, USA). The detection optodes were placed 25–30 mm above the midpoint of the eyebrow, at approximately FP1 and FP2 according to the international 10–20 electrode system. The emission optodes were laterally placed at approximately F7 and F8. The emitter-detector spacing was 50 mm to minimize contamination from the extra-cerebral circulation and maximize signal intensity. The differential pathlength factor, which accounts for the increased distance traveled by light due to scattering, is age-dependent (Duncan et al., 1996). At present, however, no data are available on the actual variation of differential pathlength factor in adults aged above 50 years. Therefore, it was set to 6.61, corresponding to age 50 (Duncan et al., 1996; Claassen et al., 2006).

### Data processing

Functional Near-Infrared Spectroscopy data were analyzed using commercially available software (Oxysoft, Artinis Medical Systems, Netherlands). Movement artefacts were kept to a minimum by instructing the participants to refrain from talking, frowning or chewing, to avoid head and body movements, and to sit as still as possible during the experiment. A moving average window of 1 s was applied to the [O_2_Hb] and [HHb] signals to filter out high-frequency noise, including noise of the heart beat frequency. The first three trials (all non-targets) of all conditions were excluded from behavioral and fNIRS data analyses to take the delay of the hemodynamic response into account and to obtain a stable hemodynamic state. The fNIRS signals were biased (set to zero) at the start of the fourth trial of each condition, that is 0-back, 1-back and 2-back. Changes of [O_2_Hb] and [HHb] were recalculated for 180 s from this point (Hoshi et al., 2003). Subsequently, mean relative values of [O_2_Hb] and [HHb] were calculated for the whole task period.

Behavioral performance was assessed by hit rate, correct rejection rate, and reaction time on target trials. Composite scores were calculated as [hits (%)/reaction time (ms) × 100] to take speed/ accuracy trade-offs into account.

### Statistical analysis

Statistical analysis was performed using IBM SPSS Statistics for Windows version 20.0 (IBM Corp., Armonk, NY, USA). Alpha was set at 0.05 for all analyses. Data are presented as mean ± SD. Shapiro-Wilk tests indicated that assumptions of normality were met. The effects of working-memory load on the composite scores were established by a repeated measures ANOVA with factor working-memory load (0-,1-,2-back).

**Figure 2:**
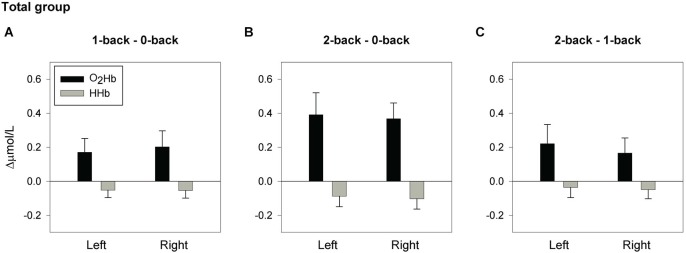
**Hemodynamic concentration changes in the total sample of older adults**. Mean (± SEM) changes of [O_2_Hb] and [HHb] in the left and right hemisphere for the spatial 1-back minus 0-back contrast **(A)**, 2-back minus 0-back contrast **(B)**, and 2-back minus 1-back contrast **(C)**.

**Figure 3:**
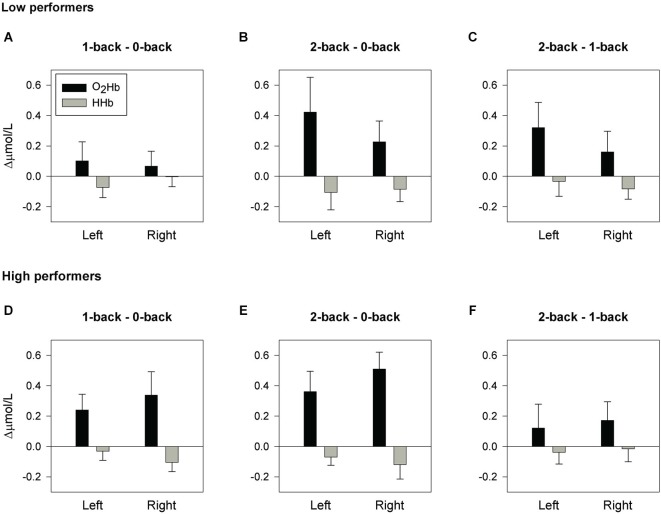
**Hemodynamic concentration changes in low and high performers**. Mean (± SEM) changes of [O_2_Hb] and [HHb] for the spatial 1-back minus 0-back contrast, 2-back minus 0-back contrast, and 2-back minus 1-back contrast.** (A)**, **(B)** and **(C)** display the results for low performers. **(D)**, **(E)** and **(F)** display the results for high performers.

[O_2_Hb] is considered to be a more robust and reproducible fNIRS parameter than [HHb] (Plichta et al., 2006). Studies have demonstrated that the fMRI BOLD response is more strongly correlated with [O_2_Hb] than with [HHb], which may be due to higher signal-to-noise ratio in [O_2_Hb] (Strangman et al., 2002; Cui et al., 2011). Therefore, taking into account the small sub-sample size, only [O_2_Hb] changes were further statistically analyzed, but results on [HHb] are presented in Figures 2, 3. For [O_2_Hb] changes, a 2 (location: left, right hemisphere) × 3 (load: 0-,1-,2-back) repeated measures ANOVA was performed. Based on the composite score for the high working-memory load condition, the group was divided by median split into nine low and nine high performers. These two groups did not significantly differ with respect to the variables age, years of education, estimated IQ, and MMSE score (see Table 1). Accordingly, a 2 (group: low, high performers) × 2 (location: left, right hemisphere) × 3 (load: 0-,1-,2-back) repeated measures ANOVA was performed. Due to violations of the sphericity assumption, Greenhouse-Geisser corrections were applied. Significant main and interaction effects were further analyzed by means of planned contrasts. Due to the small sub-sample size, effects sizes (partial eta squared; $\eta_{p}^{2}$) will be reported as well. $\eta_{p}^{2}$ ranges from 0 to 1 and it indicates the proportion of variance in the dependent variable that is attributable to the independent variable. An effect size of $\eta_{p}^{2}$ = 0.01 is considered to be small, $\eta_{p}^{2}$ = 0.06 medium, and $\eta_{p}^{2}$ = 0.14 large (Cohen, 1988). To establish the relationship between working-memory load related changes of composite scores and [O_2_Hb] changes, Pearson correlation coefficients (2-tailed) were calculated, corrected for age and years of education.

## Results

### Behavioral performance

Table 2 shows the behavioral results of the older adults during performance of the n-back tasks. Increased working-memory load led to a declined hit rate (*F*_(1.48, 25.14)_ = 6.91, *p* = 0.008; 0- vs. 1-back *p* = 0.064; 0- vs. 2-back *p* = 0.001; 1- vs. 2-back *p* = 0.088), and a declined correct rejection rate (*F*_(1.19, 20.19)_ = 19.35, *p* < 0.001; 0- vs. 1-back *p* = 0.033; 0- vs. 2-back *p* < 0.001; 1- vs. 2-back *p* = 0.001). Also, in comparison with the control condition, both low and high working-memory load led to increased reaction times on target and non-target trials (all *p*-values < 0.001). Furthermore, composite scores were negatively affected by load (*F*_(2, 34)_ = 30.63, *p* < 0.001; 0- vs. 1-back *p* = 0.001; 0- vs. 2-back *p* < 0.001; 1- vs. 2-back *p* = 0.001).

**Table 2:** **Accuracy and reaction times (Mean ± SD) for the spatial n-back tasks**.

		Total (*n* = 18)	Low performers (*n* = 9)	High performers (*n* = 9)
Hits (%)	0-back	95.8 ± 8.0	96.7 ± 6.0	94.8 ± 9.9
	1-back	91.2 ± 12.0	89.5 ± 13.4	92.8 ± 10.9
	2-back	84.0 ± 12.9	78.4 ± 11.8	89.5 ± 12.0
Correct rejections (%)	0-back	99.2 ± 2.0	99.2 ± 2.3	99.2 ± 1.6
	1-back	97.5 ± 2.5	97.2 ± 2.3	97.9 ± 2.7
	2-back	89.9 ± 9.0	88.1 ± 9.3	91.7 ± 8.8
RT target (ms)	0-back	665.9 ± 110.0	697.7 ± 104.3	634.0 ± 112.0
	1-back*	772.7 ± 148.2	842.3 ± 138.8	703.1 ± 128.4
	2-back**	1048.5 ± 333.8	1316.4 ± 229.2	780.7 ± 151.0
RT non-target (ms)	0-back	643.8 ± 87.0	665.9 ± 73.3	621.7 ± 98.0
	1-back	743.0 ± 124.6	761.2 ± 77.1	724.8 ± 162.1
	2-back**	958.5 ± 270.7	1143.4 ± 221.5	773.6 ± 172.3
Composite score	0-back	14.6 ± 2.0	14.1 ± 1.9	15.2 ± 2.1
	1-back*	12.2 ± 2.7	11.0 ± 2.8	13.4 ± 2.0
	2-back**	8.9 ± 3.3	6.2 ± 1.4	11.7 ± 2.0

**p ≤ 0.005, *p < 0.05 low performers vs. high performers.

Investigating the high and low performers separately, both groups showed a significantly decreased correct rejection rate, increased reaction times on targets and non-targets, and a decreased composite score with increased working-memory load (all *p*-values < 0.05). Hit rate declined in low performers (*p* = 0.011), but not in high performers (*p* = 0.261). Table 2 shows the statistically significant group differences on the behavioral parameters.

### fNIRS results—overall group

Figure 2 displays the mean [O_2_Hb] changes for the 1-back minus 0-back contrast, 2-back minus 0-back contrast, and 2-back minus 1-back contrast. Whole-group analysis revealed a significant main effect of load (*F*_(2,34)_ = 7.99, *p* = 0.001, $\eta_{p}^{2}$ = 0.320; 0- vs. 1-back *p* = 0.039, $\eta_{p}^{2}$ = 0.226; 0- vs. 2-back *p* = 0.002, $\eta_{p}^{2}$ = 0.443), with a trend for 1- vs. 2-back (*p* = 0.063, $\eta_{p}^{2}$ = 0.189). No significant effects were found for the factor location, or the location × load interaction, indicating bilateral activation during task performance. Further analysis confirmed a load effect in both the left fNIRS channel (*F*_(2,34)_ = 6.41, *p* = 0.004, $\eta_{p}^{2}$ = 0.274; 0- vs. 1-back, *p* = 0.050, $\eta_{p}^{2}$ = 0.208; 0- vs. 2-back *p* = 0.007, $\eta_{p}^{2}$ = 0.352) and the right fNIRS channel (*F*_(2,34)_ = 8.02, *p* = 0.001, $\eta_{p}^{2}$ = 0.321; 0- vs. 1-back *p* = 0.048, $\eta_{p}^{2}$ = 0.212; 0- vs. 2-back *p* = 0.001, $\eta_{p}^{2}$ = 0.482). Trends were found for 1- vs. 2-back (Left: *p* = 0.069, $\eta_{p}^{2}$ = 0.182; Right: *p* = 0.080, $\eta_{p}^{2}$ = 0.170).

### fNIRS results—low and high performers

A large effect size was found for the significant interaction of group × location × load (*F*_(2,32)_ = 3.55, *p* = 0.041, $\eta_{p}^{2}$ = 0.182). The interaction of group × location showed a trend towards significance and a large effect size (*F*_(1,16)_ = 3.70, *p* = 0.073, $\eta_{p}^{2}$ = 0.188). These results indicate group differences in prefrontal activation that may not be consistent across tasks and hemispheres.

The interaction of group × location was further analyzed for each individual condition. For the 0-back task and 1-back task, no significant effects of group, location, or group × location were found, indicating bilateral activation in both groups. For the 2-back task, analyses revealed a significant group × location interaction with a large effect size (*F*_(1,16)_ = 6.27, *p* = 0.023, $\eta_{p}^{2}$ = 0.282). No significant main effects of location or group were found. Further group comparisons however, showed a large effect size for the right fNIRS channel (*F*_(1,16)_ = 2.72, *p* = 0.119, $\eta_{p}^{2}$ = 0.145), which may suggest stronger activation in the high performers than in the low performers during 2-back performance. Furthermore, the effect of location showed a trend towards significance with a large effect size in low performers, indicating lower activation in the right hemisphere compared to the left hemisphere (*F*_(1,8)_ = 4.00, *p* = 0.080, $\eta_{p}^{2}$ = 0.333). In high performers, no significant effect of location was found, but the large effect size may suggest stronger activation in the right hemisphere compared to the left hemisphere during performance of the 2-back task (*F*_(1,8)_ = 2.36, *p* = 0.163, $\eta_{p}^{2}$ = 0.227).

The effects of load were analyzed for low- and high-performing elderly separately. Figure 3 shows the mean [O_2_Hb] changes for the 1-back minus 0-back contrast, 2-back minus 0-back contrast, and 2-back minus 1-back contrast for both groups. In high performers, significant load effects with a large effect size were found for the left fNIRS channel (*F*_(2,16)_ = 3.77, *p* = 0.046, $\eta_{p}^{2}$ = 0.320; 0- vs. 1-back *p* = 0.049, $\eta_{p}^{2}$ = 0.401; 0- vs. 2-back *p* = 0.028, $\eta_{p}^{2}$ = 0.475; 1- vs. 2-back *p* = 0.467, $\eta_{p}^{2}$ = 0.068) and for the right fNIRS channel (*F*_(2,16)_ = 7.86, *p* = 0.004, $\eta_{p}^{2}$ = 0.495; 0- vs. 1-back *p* = 0.060, $\eta_{p}^{2}$ = 0.374; 0- vs. 2-back *p* = 0.002, $\eta_{p}^{2}$ = 0.723; 1- vs. 2-back *p* = 0.200, $\eta_{p}^{2}$ = 0.196). In low performers, the effects of load were not statistically significant, but large effect sizes were found for the left fNIRS channel (*F*_(2,16)_ = 3.05, *p* = 0.075, $\eta_{p}^{2}$ = 0.276; 0- vs. 1-back *p* =0.444, $\eta_{p}^{2}$ = 0.075; 0- vs. 2-back *p* = 0.102, $\eta_{p}^{2}$ = 0.299; 1- vs. 2-back *p* = 0.088, $\eta_{p}^{2}$ = 0.320) and for the right fNIRS channel (*F*_(2,16)_ = 1.73, *p* = 0.209, $\eta_{p}^{2}$ = 0.178; 0- vs. 1-back *p* = 0.517, $\eta_{p}^{2}$ = 0.054; 0- vs. 2-back *p* = 0.138, $\eta_{p}^{2}$ = 0.254; 1- vs. 2-back *p* = 0.273, $\eta_{p}^{2}$ = 0.147).

### Relation behavioral performance and fNIRS

**Figure 4:**
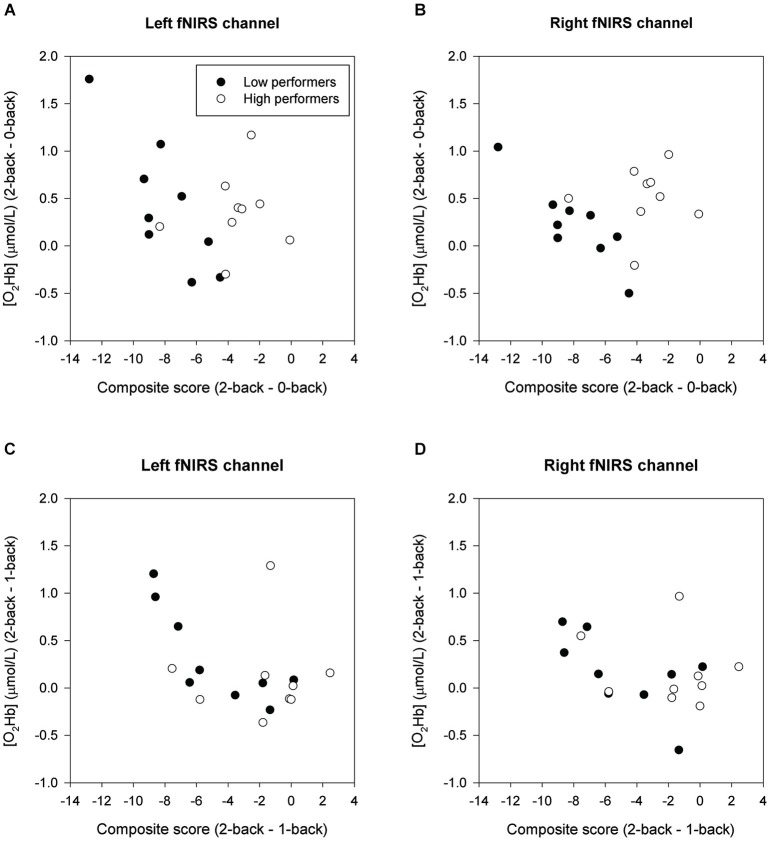
**Correlation of δComposite score and δ[O_2_Hb] in low and high performers. (A)** and **(B)** show the 2-back minus 0-back contrast. **(C)** and **(D)** show the 2-back minus 1-back contrast.

Figure 4 displays scatterplots illustrating the relationships between cognitive load-induced changes in composite score and [O_2_Hb]. Whole-group analysis demonstrated that individuals with a larger decline in composite score from the 0-back to the 2-back condition had a larger increase of [O_2_Hb] in the left fNIRS channel between these conditions (Left: *r* = −0.504, *p* = 0.046; Right: *r* = −0.158, *p* = 0.559). Individuals with a larger decline in composite score from the 1-back to the 2-back condition showed a larger increase of [O_2_Hb] in the left fNIRS channel (Left: *r* = −0.503, *p* = 0.047; Right: *r* = −0.427, *p* = 0.099).

Within the low performers group, individuals with a larger decline in composite score from the 0-back to the 2-back condition had a larger bilateral increase of [O_2_Hb] between these conditions (Left: *r* = −0.803, *p* = 0.030; Right: *r* = −0.872, *p* = 0.010). Furthermore, low performers with a larger decline in composite score from the 1-back to the 2-back condition showed a larger increase of [O_2_Hb] in the left fNIRS channel (Left: *r* = −0.856, *p* = 0.014; Right: *r* = −0.577, *p* = 0.175). For the high performers group, no significant correlations were found between load-related changes in behavioral performance and hemodynamic changes (2- back minus 0-back: Left: *r* = 0.204, *p* = 0.661; Right: *r* = 0.102, *p* = 0.827; 2-back minus 1-back: Left: *r* = 0.074, *p* = 0.875; Right: *r* = −0.110, *p* = 0.814).

## Discussion

In the present study, fNIRS was used to investigate possibly compensatory brain-behavior mechanisms at older age, by assessing prefrontal activation in low- and high-performing older adults during spatial working-memory performance. As expected, increased working-memory load led to increased prefrontal activation and decreased behavioral performance. Results revealed an interaction between performance level and hemispheric activation, which suggests stronger right prefrontal activation in high performers in comparison to low performers under high cognitive demand. Furthermore, in low performers, a larger decline in task performance with increasing working-memory load condition was associated with a larger bilateral upregulation of prefrontal activation. In high performers, no correlation between behavioral performance and prefrontal activation was found. Taken together, these results support the view that prefrontal activation may not only be modulated by working-memory load, but may also be related to performance level.

Whole-group analysis revealed an upregulation of left and right prefrontal activation with increasing spatial working-memory load. This pattern of bilateral recruitment is in accordance with the HAROLD model (Cabeza, 2002). Previous neuroimaging studies in the visual-spatial working-memory domain (Petrella et al., 2005; Holtzer et al., 2009; Nagel et al., 2009; Toepper et al., 2014), and the verbal working-memory domain (Mattay et al., 2006; Nyberg et al., 2009; Cappell et al., 2010; Prakash et al., 2012; Sala-Llonch et al., 2012; Vermeij et al., 2012) have shown sensitivity of prefrontal activation to task demand in older adults. However, the shape of the dose-response curve varies among studies; in the current study we found an increase of prefrontal activation up to 2-back in our study, while, for example, Mattay et al. (2006) found a consistent decrease with load in their n-back study, and Heinzel et al. (2014) found a tendency towards an inverted U-shape. This variation may depend on factors such as task design, population and task difficulty (Stern et al., 2012). These findings emphasize the need to take behavioral performance level into account when interpreting and comparing neuroimaging data.

In the current study, activation patterns of high and low performers were compared. We found a significant interaction between performance level and hemispheric activation, indicating that high performers more strongly activated the right prefrontal cortex under high working-memory load than low performers did. Direct comparison of left and right hemispheric activation within each group did not result in significant differences, but the large effect sizes may suggest that low performers show decreased activation in the right hemisphere in comparison to the left hemisphere under high cognitive demand, while the opposite pattern was found in high performers. These performance-specific findings may indicate that the commonly observed bilateral prefrontal activation pattern, which we also found in our whole-group analysis, may in fact obscure the heterogeneity in activation patterns in older adults and may lead to invalid generalizations.

A prior study on the hemodynamic response to a spatial working-memory challenge (Nagel et al., 2009) found that the dose-response curves of high-performing older adults resembled those of young adults in most investigated regions of interest, showing an increase of activation with load. In contrast, low-performing older adults showed a drop in activation at the highest level of working-memory load. The interaction between performance level and load was present in right dorsolateral prefrontal cortex, but not in the left dorsolateral prefrontal cortex. Sala-Llonch et al. (2012) demonstrated that high-performing older adults showed stronger activation of the right inferior gyrus than low-performing older adults during performance of a verbal 2-back task. In comparison to young adults, high-performing older adults showed increased bilateral frontal activation and increased connectivity in the right frontoparietal task-related network. Moreover, high performers recruited frontal areas involved in the default network, indicating that recruitment of task-unrelated resources might be part of a successful compensatory mechanism of the aging brain. These results in combination with our findings suggest that recruitment of the right prefrontal cortex may be beneficial for working-memory performance in older adults.

We further explored the brain-behavior relationship and found that low performers who demonstrated a larger load-induced decline in behavioral performance showed a larger load-induced increase in bilateral prefrontal activation. In high performers, we did not find an association between behavioral performance and prefrontal activation. The lack of significant correlations may be due to the limited range of accuracy scores. Although behavioral performance significantly declined with increasing load in this group, performance was on average very high. Alternatively, the sub-sample size may not have been large enough to detect significant associations, which is a limitation of this study.

Previous neuroimaging studies on working-memory performance in older adults showed mixed results on brain-behavior correlations. Nagel et al. (2011) found that BOLD signal changes, induced by increasing verbal working-memory load, were positively correlated with accuracy scores during 3-back performance in the left and right premotor cortex and right posterior parietal cortex, and, at trend level, in the left and right dorsolateral prefrontal cortex. Podell et al. (2012) reported that caudate activation was associated with improved accuracy on a working-memory task, and that ventrolateral prefrontal activation was associated with shorter reaction times. Nagel et al. (2009) reported a positive correlation between BOLD signal changes in the left premotor cortex and accuracy scores on a spatial working-memory load task. Furthermore, Toepper et al. (2014) showed that activation in the dorsolateral prefrontal cortex was positively correlated with the numbers of errors at a low level of spatial working-memory load. No significant correlations were found for higher levels of working-memory load. Several other studies however, failed to find working-memory-related brain-behavior correlations in older adults, possibly in part due to near-ceiling accuracy levels (Emery et al., 2008; Holtzer et al., 2009; Cappell et al., 2010; Piefke et al., 2012). Since we were able to establish significant correlations in a relatively small sub-sample, we argue that the fNIRS [O_2_Hb] signal might be a more sensitive parameter for detecting brain-behavior associations than the fMRI BOLD signal.

Since our aim was to gain insight into the possibly compensatory brain-behavior mechanisms at older age, we did not include a group of young adults to examine age-related changes in prefrontal activation. However, our results may be congruent with the CRUNCH hypothesis that prefrontal over-recruitment may reflect an age-invariant compensatory mechanism (Reuter-Lorenz and Cappell, 2008). A significant interaction of performance level and hemispheric activation indicated that high performers were better able than the low performers to keep the right prefrontal cortex engaged at high working-memory load. Low performers may have reached the limit of available neural resources, while high performers may have been able to recruit more neural resources. Hence, recruitment of the right prefrontal cortex might contribute to successful working-memory performance in older adults (Sala-Llonch et al., 2012). In contrast, the negative correlation between load-induced changes in activation and performance that was observed in low performers may point towards declined neural efficiency or unsuccessful compensation rather than successful compensation (Cabeza and Dennis, 2012).

The term “compensation” has been under debate. According to some researchers, compensation reflects the recruitment by older adults of the same brain regions that are recruited by young adults in response to increasing task demand. Older adults may need to recruit these resources at lower levels of task demand, but the cognitive operations that contribute to task performance are age invariant (Reuter-Lorenz and Cappell, 2008; Cappell et al., 2010). According to other researchers, the term “compensation” should only be used in case older adults show recruitment of brain regions that are not recruited by younger adults. Moreover, engagement of these regions should be directly correlated to a better performance in older adults, but not be related to the performance in younger adults (Stern, 2002). Since we only measured activation in the prefrontal cortex, with limited spatial resolution, we are not be able to evaluate whether or not the older adults showed a reorganization of neurocognitive networks. However, in previous studies we observed cognitive load-dependent activation in the same brain region in a group of younger adults (Vermeij et al., 2012, 2014). This would support the age-invariant view of compensatory recruitment.

Another limitation in this study was that the order of conditions was ascending (0-back, 1-back, 2-back), as is common in neuropsychological assessment of working-memory span, instead of counterbalanced. Given the finding that prefrontal activation increased with load, we consider it unlikely that the order of presentation may have confounded the results.

Examination of the relationship between possibly compensatory brain activation and behavioral outcomes may provide starting points for the development and evaluation of cognitive training programs. Heinzel et al. (2014) reported that the BOLD response pattern and accuracy during n-back performance were predictive of behavioral training gain in older adults. Future research should aim to establish the plasticity of prefrontal compensatory mechanisms, by studying healthy older adults as well as those who are in a preclinical stage of dementia. Cognitive training programs are attractive, especially to those who suffer from cognitive problems, but to date it is unknown how interindividual neurocognitive differences contribute to training success.

To conclude, we observed performance-related differences in prefrontal activation in older adults during working-memory performance. Additional recruitment of the right prefrontal cortex may be beneficial for performance when task demands are high. However, an increase in bilateral prefrontal activation with cognitive load may not always be compensatory. Therefore, for the interpretation of neuroimaging data, individual behavioral performance should be taken into account to be able to distinguish successful and unsuccessful compensation or declined neural efficiency.

## Conflict of interest statement

The authors declare that the research was conducted in the absence of any commercial or financial relationships that could be construed as a potential conflict of interest.
